# Chemo-preventive effects and antitumorigenic mechanisms of beer and nonalcoholic beer toward 4-(methylnitrosamino)-1-(3-pyridyl)-1-butanone (NNK) - induced lung tumorigenesis in A/J mice

**DOI:** 10.1186/s41021-023-00276-3

**Published:** 2023-06-07

**Authors:** Jun Takata, Katsuyuki Kiura, Takamasa Nakasuka, Atsuko Hirabae, Sakae Arimoto-Kobayashi

**Affiliations:** 1 Graduate School of Medicine, Dentistry and Pharmaceutical Sciences, Okayama University, Okayama, 700-8530 Japan; 2 Department of Allergy and Respiratory Medicine, Okayama University Hospital, Okayama, 700-8530 Japan

**Keywords:** Anti-mutagenesis, Signal transduction, Lung tumorigenesis, DNA methylation, Tobacco-specific nitrosamine, Glycine betaine

## Abstract

We investigated the chemopreventive effects of beer, nonalcoholic beers (NABs), and beer-components (glycine betaine (GB)) on NNK-induced lung tumorigenesis in A/J mice, and the possible mechanisms underlying the antitumorigenic effects of beer, NABs, and beer-components. Beer, NABs, and GB reduced NNK-induced lung tumorigenesis. We investigated the antimutagenicity of beer, NABs and beer-components (GB and pseudouridine (PU)) toward the mutagenicity of 1-methyl-3-nitro-1-nitrosoguanidine (MNNG) and 4-(methylnitrosamino)-1-(3-pyridyl)-1-butanone (NNK). Beer, NABs, and beer components were antimutagenic toward MNNG and NNK in the Ames test using *S. typhimurium* TA1535. In contrast, MNNG and NNK mutagenicity detected in *S. typhimurium* YG7108, a strain lacking *O*^6^-methylguanine DNA methyltransferases (*ogt*_ST_ and *ada*_ST_) did not decrease in the presence of beer, NABs, or beer components, suggesting that they may mediate its antimutagenic effect by enhancing DNA damage repair. Phosphorylation of Akt and STAT3, with or without epidermal growth factor stimulation, in lung epithelial-like A549 cells were significantly decreased following beer, NABs, GB and PU. They targeted both the initiation and growth/progression steps of carcinogenesis, specifically via antimutagenesis, stimulation of alkyl DNA-adduct repair, and suppression of Akt- and STAT3- mediated growth signaling. GB and PU may contribute, in part, to the biological effects of beer and NABs via the suppression of Akt and STAT3 phosphorylation.

## Introduction

Lung cancer is the most common cause of cancer-related deaths [1]. The death rate of lung cancer continues to increase in Japan [2]. Tobacco smoke is one of the biggest causes of cancer development, particularly lung cancer [3, 4]. Regarding the harmful effects of tumor-promoting tobacco carcinogens, 4-(methylnitrosamino)-1-(3-pyridyl)-1-butanone (NNK), a nitrosamine ketone derived from nicotine, has been implicated. Although many tobacco agents have been implicated in the development of lung tumors, the potent effects of NNK remain unparalleled [5]. NNK induced extensive DNA damage in rat and mouse target tissues. The types of damage include methylation, pyridyl oxobutylation, oxidation, and chain breaks. Metabolic activation of NNK results in DNA methylation and pyridyl oxobutylation by α-hydroxylation [6]. All commercial tobacco products contain NNK and molecular epidemiological studies have revealed a relationship between NNK and lung cancer has been revealed in molecular epidemiological studies [7]. Experimentally, lung tumors can be easily induced in mice, rats and hamsters by NNK [8]. NNK plays an important role in the development of lung cancer in smokers.

Beer is one of the most consumed alcoholic beverages in the world and is rich in nutrients such as carbohydrates, amino acids, minerals, vitamins, and other compounds such as polyphenols. Hops are one of the raw materials for beer, which serves as an important source of phenolic compounds [9]. Approximately 30% of beer-derived polyphenols originate from hops and 70–80% from malt [10]. In our early study, beer inhibited DNA adduct formation in liver and lungs in mice treated with heterocyclic amines [11], and 2-amino-1-methyl-6-phenylimidazo[4,5-b]pyridine (PhIP)-induced mammary carcinogenesis in female rats [12]. Gerhäuser [13] reviewed that beer constituents have potential as chemopreventive agents against cancer.

Recently, nonalcoholic beers (NABs) have been developed, and the size of this market is increasing because of the increasing adoption of healthier lifestyles and awareness of the benefits of alcohol-free beverages. Nonalcoholic beer can be produced in various ways, including the removal of alcohol from beer after production or the use of special yeast to avoid the production of alcohol during brewing [14]. Depending on the manufacturing methods, ingredients and tastes differ.

In our previous study, we identified pseudouridine (PU) and glycine betaine (GB) as active components in beer responsible for antimutagenicity [15, 16]. GB was reported to protect against high-fat-diet-induced liver injury and alcoholic liver injury [17, 18]. PU is a modified base in RNA and is found in all species, but little is known about the physiological activity of PU [19].

In the present study, we investigated the antimutagenic activity of NABs and beer components (GB and PU) toward the alkylating agent 1-methyl-3-nitro-1-nitrosoguanidine (MNNG) and NNK, and its mechanisms. We also investigated the chemopreventive effects of beer, NABs, and GB on NNK-induced lung tumorigenesis in A/J mice, and the molecular mechanism of the antitumor effect and suppression of tumor growth in the presence of beer and beer components.

## Materials and methods

### Materials

Commercial beer, low alcoholic beer (hereinafter referred to as LAB; sample code C), and nonalcoholic beer (hereinafter referred to as NAB; sample codes A, B, and D–F) were purchased from local stores in Okayama City. The alcohol content of the beer sample was 5%, that of LAB was 0.5%, and those of NABs were 0%. All the samples except NAB(B) were produced in Japan, and NAB(B) was imported from Germany. For the Ames test, beer, LAB, and NABs were sterilized by filtration. For further experiments, the beer was freeze-dried and the alcohol was removed. NAB(A) and NAB(B) were freeze-dried. The residues were dissolved in water to one-tenth the original volume (hereafter referred to as the 10-fold concentrate). The 10-fold concentrates were stored at -20 °C and diluted just before use. GB and PU were purchased from FUJIFILM Wako Pure Chemical Co. (Osaka, Japan). NNK were purchased from Toronto Research Chemicals (North York, ON, Canada), MNNG was from Nacalai Tesque (Kyoto, Japan), and 3-(4,5-Dimethyl-2-thiazolyl)-2,5-diphenyl-2H-tetrazolium bromide (MTT) was from Dojindo Laboratories (Kumamoto, Japan). For metabolic activation, the supernatant fraction of rat liver homogenate (S9) prepared using phenobarbital and 5,6-benzoflavone was obtained from FUJIFILM Wako Pure Chemical Co. Akt (pan) (C67E7) rabbit mAb (#4691), phospho-Akt (Ser473) (D9E) rabbit mAb (#4060), STAT3 (79D7) rabbit mAb (#4904), phospho-STAT3 (Tyr705) (D3A7) XP rabbit mAb (#9145), LY294002 (#9901) monoclonal antibodies (mAbs) was purchased from CST, Japan (Tokyo, Japan), and Stattic (ab120952) from Abcam (Cambridge, UK). All the other reagents were purchased from commercial sources.

*Salmonella enterica* subspecies I, serovar Typhimurium (*Salmonella typhimurium*) strain TA1535 [*hisG46 ΔuvrB gal bio chl1005 rfa1001*] was a gift from Dr. B. N. Ames of the University of California, Berkeley [20], and *S. typhimurium* YG7108 [*hisG46 ΔuvrB gal bio chl1005 rfa1001 Δada*_*st*_*::Km*^*r*^* Δogt*_*st*_*::Cm*^*r*^], a strain lacking *O*^6^-methylguanine DNA methyltransferases, was gifted from Dr. M. Yamada of the National Institute of Health Sciences [21]. Human lung epithelial-like cell line A549 from lung carcinoma (hereinafter referred to as A549 cells), which refers to ATCC CCL185, was provided by RIKEN BRC through the National BioResource Project of the MEXT/AMED, Japan (Tsukuba, Japan).

Quantitative analysis of GB and PU in NAB was outsourced to the Saitama Prefectural Industrial Technology Center. Analyses were performed using Agilent Technologies 1260 Infinity II HPLC and 6120 single quadrupole mass spectrometers (Agilent Technologies Japan, Ltd., Tokyo). The GB content was 92 µM in NAB(A) and 290 µM in NAB(B). The PU contents were 5.2 µM in NAB(A) and 22 µM in NAB(B).

### Animals

Mice (A/J Jms Slc female, 3-weeks old) were purchased from Japan SLC, Inc. (Hamamatsu, Japan). Five mice were housed in animal compartments for each cage and randomly divided into treatment groups at least one week before the start of the experiment. Murine chow (MF powder, Oriental Yeast Co., Ltd., Tokyo, Japan) was mixed with water or an experimental solution to produce feed-lumps. The mice had free access to feed-lumps and water, and were maintained with optimal air exchange and a 12-h light/12-h dark cycle at a constant room temperature of 20 °C. All the experiments were conducted in accordance with the Guidelines for Animal Experiments of the Okayama University Advanced Science Research Center (permission no. OKU-2018028, 2018030, 2019671, 2021460, 2021461, 2022299, and 2022336) based on the Act on Welfare and Management of Animals (Act of Japan, No. 105 of October 1, 1973, and Amendment of Act No. 68 of 2005) and Standards Relating to the Care, Management, and Alleviation of Pain and Distress of Laboratory Animals (Notice of the Ministry of the Environment No. 88 of 2006).

### Antimutagenicity test

The effects of beer, LAB, NABs, ethanol, PU and GB on MNNG-induced mutagenicity were investigated using the Ames test [20] with *S. typhimurium* TA1535 in the absence of rat liver homogenate (hereafter referred to as -S9) as previously described [22]. Beer, LAB, NABs, PU, and GB were filter-sterilized and used in the assay. The inhibitory effects of beer, NAB(A), NAB(B), PU, and GB on the mutagenicity of MNNG in *S. typhimurium* YG7108 (-S9) were also investigated. The amounts of beer, LAB, and NAB used for the assay calculated as microliter equivalents of the original volume (µL eq.) of the original beer, LAB, and NABs, respectively. The effects of beer, NAB(A), NAB(B), PU and GB on NNK-induced mutagenicity were also investigated using the Ames test with *S. typhimurium* TA1535 and YG7108 in the presence of rat liver homogenate for metabolic activation (hereafter referred to as + S9). Other procedures were as described above. The experiments were performed in triplicates. All plates were examined with a stereo microscope whether background lawn was impaired.

The mutagenic activity (%) was derived as follows:

$$100\;\times\;\left[\left(\mathrm{revertants}\;\mathrm{in}\;\mathrm{the}\;\mathrm{presence}\;\mathrm{of}\;\mathrm{beer},\;\mathrm{LAB},\;\mathrm{NAB},\;\mathrm{PU},\;\mathrm{or}\;\mathrm{GB}\right)\;-\;\left(\mathrm{spontaneous}\;\mathrm{revertants}\right)\right]\;/\;\left[\left(\mathrm{revertants}\;\mathrm{in}\;\mathrm{the}\;\mathrm{absence}\;\mathrm{of}\;\mathrm{beer},\;\mathrm{LAB},\;\mathrm{NAB},\;\mathrm{PU},\;\mathrm{or}\;\mathrm{GB}\right)\;-\;\left(\mathrm{spontaneous}\;\mathrm{revertants}\right)\right].$$

### Anti-tumorigenesis study in mice with beer, GB and NABs

NNK-induced tumorigenesis experiments were conducted according to previous studies [23]. In experiment 1, mice (A/J, 4 weeks of age, mean weight 10.1 ± 1.3 g) were divided into 6 groups of 10–15 animals each (groups I – VI). Freeze-dried beer was diluted with water to half the volume of the original beer (hereinafter referred to as 200% beer). The feed-lumps were produced by mixing murine-chow powder with water, 1.5 M GB or 200% beer (hereinafter referred to as water, GB or beer diet, respectively). Mice of groups I and IV received a water diet throughout the experiment. Mice in groups II and V received a GB diet, and mice in groups III and VI received a beer diet from 4 weeks of age until the end of the experiment. Tumor induction was achieved by a single intraperitoneal (i.p.) injection of 100 mM NNK in 0.1 ml saline at 8 weeks of age in groups I, II, and III. Mice of groups IV, V, and VI were injected with 0.1 mL of normal saline as a substitute for NNK (control). The mice were euthanized at the age of 30 weeks.

In experiment 2, mice (A/J, 4 weeks old, 10.4 ± 1.3 g) were divided into 5 groups (groups VII-XII) of 10–15 animals each. Prior to the experiment, the two nonalcoholic beers (NAB (A) and NAB (B)) were freeze-dried and dissolved in water to half the volume of the original NAB (hereinafter referred to as 200% NAB (A) and 200% NAB (B)). The feed-lumps were produced by mixing murine-chow powder with water, 200% NAB(A) or 200% NAB(B) (hereafter referred to as the water, NAB(A), or NAB(B) diets, respectively). At 8 weeks of age, mice in groups VII, VIII, and IX were injected with 100 mM NNK in 0.1 ml saline, while mice in groups X, XI, and XII were given a single i.p. injection of 0.1 mL saline. The mice in groups VII and X received a water diet throughout the experiment. Mice in groups VIII and XI received the NAB(A) diet, and mice in groups IX and XII received the NAB(B) diet from 4 weeks of age until the end of the experiments. The mice were euthanized at the age of 30 weeks. In both experiments, the number of surface lung nodules in the left lung lobe was counted using a loupe and a Digital Caliper (AS ONE Corporation, Osaka, Japan). Counted nodules were with a diameter of > 1 mm. The left lung lobes were fixed, the sections were stained with hematoxylin and eosin (HE), and pathological analysis was performed.

### Cell viability measurement

Cell viability was measured using the MTT assay to determine the concentration of beer, NAB, PU and GB for the studies of phosphorylation effect as previously described [24]. Briefly, A549 cells (1 × 10^4^ cells, 90 μL/well) were seeded in 96-well plates. After 24 h incubation (37 °C, 5% CO_2_), 10 μL of beer (finally, 10–50%beer), NAB (finally, 60–100%NAB), PU (finally, 0.001 –10 mM) or GB (finally, 200–400 mM) was added to each well. After incubation for 24 h, MTT solution (10 μL/well) was added and incubated for 2 h. The MTT-containing medium was removed, and for dissolving the formazan complexes, dimethyl sulfoxide (DMSO) was added to the wells. The optical density of the cells was measured, and optical density of the control (untreated) cells was set at 100% viability.

### Effects of phosphorylation of STAT3 and Akt in A549 cells

The effects of phosphorylation of STAT3 and Akt in A549 cells was determined as previously described [24]. Briefly, A549 cells were cultured in dishes (35 mm in diameter) for 24 h, the 0.3 mL of beer (finally, 40%beer), NAB (finally, 70%NAB), PU (finally, 1 mM), or GB (finally, 250 mM) dissolved in Dulbecco's Modified Eagle Medium (DMEM) were added, and the cells were incubated for 24 h. For the negative control (NC), A549 cells were cultured for 24 h and 0.3 mL DMEM (without beer, NAB, PU nor GB) was added. For the positive control (PC), A549 cells were cultured for 24 h and a positive kinase-inhibitor control (stattic (final concentration [conc.], 20 µM) for STAT3, and LY294002 (final conc., 50 µM) for Akt) (without beer, NAB, PU, or GB) were added to the PC dish 1 h before termination of incubation. For experiments stimulating epidermal growth factor (EGF), EGF (final conc., 100 ng/mL) was added 10 min before the termination of incubation. In the experiments stimulating interleukin-6 (IL-6), IL-6 (final conc., 5 µg/mL) was added 30 min before the termination of incubation. After incubation, the cells were harvested and washed by phosphate-buffered saline (PBS(-)). Then, cells were lysed in a cell lysis buffer (CLB) and were disrupted by ultrasound to obtain protein solutions. Protein concentrations were quantified by a DC Protein Assay Kit (Bio-Rad, Hercules, CA, USA). For western blotting, protein which were equal concentrations were electrophoretically separated on a sodium-dodecyl sulfate (12%) gel and transferred onto polyvinylidene difluoride membranes. The membranes were blocked with 5% skim milk/Tris-buffered saline containing 0.1% Tween 20 (TBST) for 1 h and probed with specific antibodies at 4 °C for 16 h. After washing the primary antibodies using TBST, the membranes were incubated with the appropriate horseradish peroxidase-conjugated secondary antibody (1:10,000) for 1 h at room temperature (15–30 °C). The protein bands were detected by Immostar® LD (Fujifilm Wako Pure Chemical, Osaka, Japan) and the biomolecular imager ImageQuant LAS 4000 (GE Healthcare Bio-Sciences AB, Uppsala, Sweden). For the loading controls, α-Tubulin for STAT3 and β-actin for Akt were used.

### Statistical analyses

For each data point as indicated in each figure, data are expressed as means ± standard deviation. Statistical significance was set at *P *< 0.05. Statistical analyses were performed by KaleidaGraph (Synergy Software, Reading, PA, USA) and Excel statistics (SSRI Co. Ltd., Tokyo, Japan).

## Results

### Antimutagenicity of beer, LAB, and NABs

**Fig. 1:**
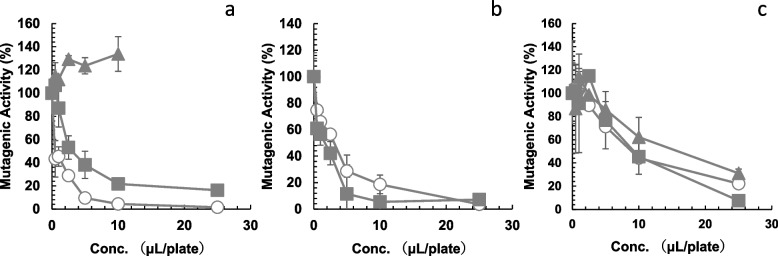
Effect of beer (**a**, circle), low alcoholic beer (LAB) (**a**, square), ethanol (**a**, triangle), NAB(A) (**b**, circle), NAB(B) (**b**, square), NAB(D) (**c**, circle), NAB(E) (**c**, square), and NAB(F) (**c**, triangle) on the mutagenicity of 1-methyl-3-nitro-1-nitrosoguanidine (MNNG), respectively. Antimutagenicity was assayed with the Ames test using *Salmonella typhimurium* TA1535 (-S9). The experiment was repeated twice and standard deviation (SD) is indicated with the bar (*n* = 3). NAB: nonalcoholic beer

The antimutagenic properties of beer, LAB, and various NABs against MNNG were investigated using the Ames test with *S. typhimurium* TA1535 without metabolic activation (-S9). The results are shown in Fig. 1. The number of His^+^ revertants per plate found in the absence of beer, LAB, NABs, GB, or PU was 2,620 ± 178 for 2.5 nmol of MNNG (TA1535, -S9) (positive control). The number of spontaneous revertants/plate (negative control) for *S. typhimurium* TA1535 (-S9) was 6.3 ± 1.3. The numbers of His + revertants per plate found for 100 µL of samples were 10.8 ± 0.96 for beer, 8.5 ± 2.5 for NAB(A), 11.5 ± 2.6 for NAB(B), 12.5 ± 5.0 for NAB(D), 9.3 ± 3.3 for NAB(E), 8.3 ± 4.5 for NAB(F), and 10.5 ± 4.4 for LAB assayed with TA1535 (-S9). Beer, LAB, and NABs did not show mutagenic activity toward *S. typhimurium* TA1535 (-S9). We examined all plates with a microscope and confirmed background lawn was not impaired and no cytotoxicity was observed. The mutagenicity of MNNG detected using TA1535 was significantly reduced in the presence of beer (Fig. 1a, circle) and LAB (Fig. 1a, square). Ethanol showed no antimutagenic activity toward MNNG (Fig. 1a, triangle). The mutagenicity of MNNG detected using TA1535 was also significantly reduced in the presence of NAB(A) (Fig. 1b, circle), NAB(B) (Fig. 1b, square) and NAB(D)–NAB(F) (Fig. 1c). Antimutagenic activities differed between the samples. The amount of sample required for 50% inhibition (ID_50_) of MNNG mutagenicity detected with TA1535 is summarized in Table 1. As ID_50_ of NAB(B) and NAB(A) were the lowest compared with those of NAB(D-F) and LAB, we selected NAB(A) and NAB(B) for further investigation.

**Table 1:** ID_50_ for antimutagenicity toward MNNG and main ingredients for beer and nonalcoholic beer

Beer	ID_50_ (µL/plate)	Alcohol conc	Raw materials^a^
Beer	0.5	5%	malt, hop, rice, corn, starch
LAB(C)	3.5	0.5%	malt, starch, malt extract, hop, barley
NAB(A)	3.1	0%	malt, barley, yeast fermented extract, hop, soda
NAB(B)	1.6	0%	malt, hop, yeast
NAB(D)	8.0	0%	malt, syrup, dietary fiber, rice fermented extract, hop
NAB(E)	9.0	0%	malt, hop, soda, spices
NAB(F)	15.8	0%	dietary fiber, soy peptides, hop, soda

^a^Indicated in each surface of the can

**Fig. 2:**
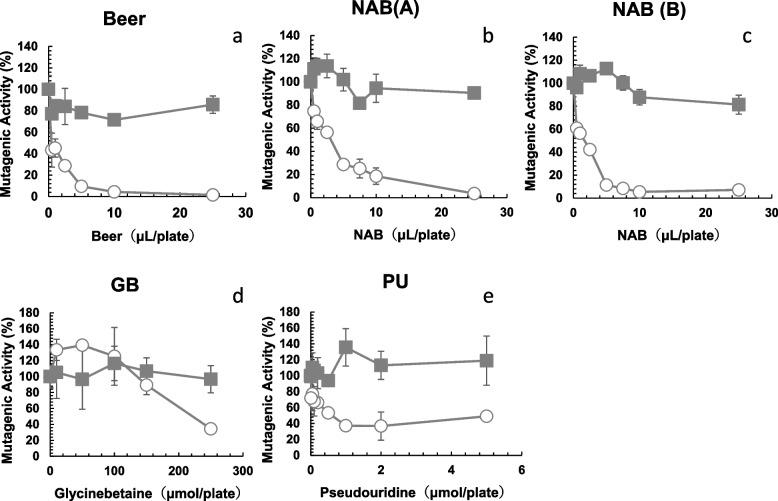
Comparison of antimutagenicity of beer (**a**), NAB(A) (**b**), NAB(B) (**c**), glycine betaine (GB) (**d**), and pseudouridine (PU) (**e**) assayed with the Ames test using *Salmonella typhimurium* TA1535 (circle) and YG7108 (square) without metabolic activation (-S9) toward MNNG. The experiment was repeated twice and standard deviation (SD) is indicated with the bar (*n* = 3). NAB: nonalcoholic beer

Alkylating agents, including MNNG, initiate their actions at the DNA level by forming alkyl DNA adducts, such as *O*^6^-alkylguanine DNA adducts. If these adducts are not removed, they mispair with the wrong base during DNA replication, resulting in mutation. *O*^6^-methylguanine DNA methyltransferases play a critical role in MNNG mutations. Therefore, we investigated the anti-mutagenic activities of samples detected with the strain deficient in *O*^6^-methylguanine DNA methyltransferase, YG7108, compared to those of the proficient strain TA1535. The number of His^+^ revertants per plate found in the absence of samples was 1,732 ± 230 for 0.25 nmol of MNNG (YG7108, -S9). The number of spontaneous revertants/plate (negative control) of *S. typhimurium* YG7108 (-S9) was 8.3 ± 5.0. The numbers of His^+^ revertants per plate found for 100 µL of samples were 16.5 ± 6.4 for beer, 9.3 ± 1.9 for NAB(A), 12.5 ± 5.0 for NAB(B) assayed with YG7108 (-S9). The numbers of His^+^ revertants per plate found for 250 µmol of GB was 2.5 ± 0.7 (TA1535, -S9) and 25.5 ± 4.0 (YG7108, -S9); and for 5 µmol of PU was 5.0 ± 1.4 (TA1535, -S9) and 10.7 ± 2.5 (YG7108, -S9). Beer, NAB(A), NAB(B), GB and PU did not show mutagenic activity toward *S. typhimurium* YG7108 (-S9). In contrast to the results obtained using TA1535 (Fig. 1 and Fig. 2a-c, circle), the MNNG mutagenicity detected with YG7108 did not decrease in the presence of beer (Fig. 2a, square), NAB(A) (Fig. 2b, square), or NAB(B) (Fig. 2c, square).

The constituents in beer, GB, and PU, inhibited the mutagenicity of MNNG, as detected using TA1535 (Fig. 2d,e, circle). In contrast, MNNG mutagenicity detected in *S. typhimurium* YG7108 was hardly reduced in the presence of GB or PU (Fig. 2d,e, square). ID_50_ of MNNG mutagenicity detected with *S. typhimurium* TA1535 was 220 µmole/plate (i.e. 25.8 mg/plate) for GB, and 0.50 µmole/plate (i.e. 0.122 mg/plate) for PU.

**Fig. 3:**
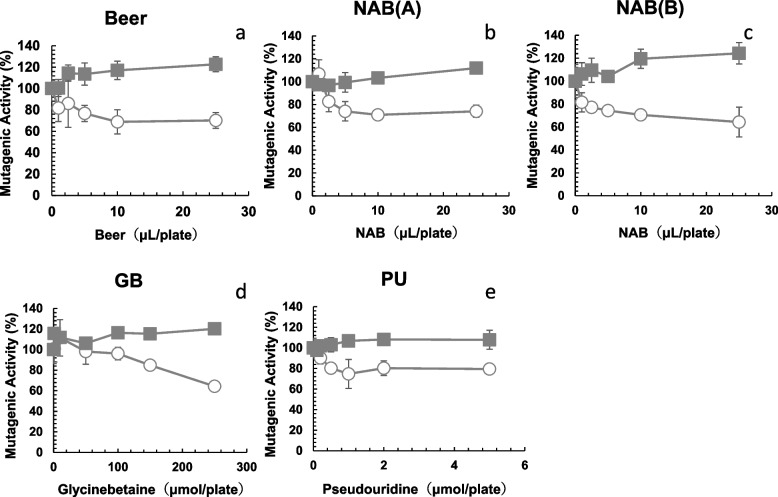
Comparison of antimutagenicity of beer (**a**), NAB(A) (**b**), NAB(B) (**c**), glycine betaine (GB) (**d**), and pseudouridine (PU) (**e**) toward NNK assayed with the Ames test using *Salmonella typhimurium* TA1535 (circle) and YG7108 (square) in the presence of metabolic activation (+ S9). The experiment was repeated twice and standard deviation (SD) is indicated with the bar (*n* = 3). NAB: nonalcoholic beer

The mutagenicity of NNK detected using TA1535 was also significantly reduced in the presence of beer (Fig. 3a, circle), NAB(A) (Fig. 3b, circle), NAB(B) (Fig. 3c, circle), GB (Fig. 3d, circle) and PU (Fig. 3e, circle). The number of His^+^ revertants per plate found in the absence of beer, LAB, NABs, GB, or PU was 556 ± 40 for 2.0 µmol of NNK (TA1535, + S9) (positive control). The number of spontaneous revertants/plate (negative control) of *S. typhimurium* TA1535 (+ S9) was 7.3 ± 0.5, and that of *S. typhimurium* YG7108 (+ S9) was 14.3 ± 1.7. The numbers of His^+^ revertants per plate found for 100 µL of samples assayed with TA1535 (+ S9) were 11.3 ± 7.7 for beer, 10.7 ± 5.2 for NAB(A), 7.3 ± 2.1 for NAB(B) and those assayed with YG7108 (+ S9) were 10.0 ± 0.8 for beer, 7.7 ± 3.9 for NAB(A), 9.0 ± 2.9 for NAB(B). The numbers of His^+^ revertants per plate found for 250 µmol of GB was 5.6 ± 1.9 (TA1535, + S9) and 8.0 ± 2.1 (YG7108, + S9); and for 5 µmol of PU was 10.6 ± 2.4 (TA1535, + S9) and 9.0 ± 1.4 (YG7108, + S9). Beer, NAB(A), NAB(B), GB and PU did not show mutagenic activity toward *S. typhimurium* TA1535 (+ S9) and YG7108 (+ S9). We investigated the anti-mutagenic activities of samples detected with YG7108, compared to those of TA1535. The number of His^+^ revertants per plate found in the absence of beer, LAB, NABs, GB, or PU was 2583 ± 38 for 100 nmol of NNK (YG7108, -S9) (positive control). In contrast to the results obtained using TA1535 (Fig. 3a-e, circle), the NNK mutagenicity detected with YG7108 did not decrease in the presence of beer (Fig. 3a, square), NAB(A) (Fig. 3b, square), NAB(B) (Fig. 3c, square), GB (Fig. 3d, square) or PU (Fig. 3e, square). All plates were examined with a stereo microscope and again confirmed background lawn was not impaired and no cytotoxicity was observed.

### Anti-tumorigenesis study with beer, GB, and NABs

In experiments 1 and 2, no noticeable clinical signs of illness were observed in mice throughout the anti-tumorigenesis study (data not shown). No significant differences in food intake were observed among groups I–XII.

Experiment 1, average bodyweight (g) of mice in groups I–VI at 30 weeks of age are shown in Table 2. The average body weight of the mice in group VI was significantly higher than that of the mice in the other groups. No significant differences in the average body weights of the mice were found among groups I–V. NNK-injected mice in group I (PC) developed a significant number of nodules on the surface of the lungs (2.13 ± 1.19, significantly different from group IV, *P* < 0.05) (Table 2). The number of nodules per mouse (0.93 ± 0 0.80) in group II, which was administered NNK and GB, was significantly decreased compared to that in group I, and the average nodule size was significantly smaller than that in group I (Table 2). The number of nodules per mouse (0.73 ± 0.88) in group III, which was administered NNK and beer, was significantly reduced compared to that in group I (Table 2). No mice in groups IV–VI developed lung nodules following the oral administration of GB or beer.

**Table 2:** Incidence and average size of lung nodules of the left lung lobe in mice

Group	Treatment NNK or saline + diet	n	Average body weight (g) at 30 wks	% of mice with nodules	No. of nodules per mouse	Average size of nodules (mm)
Exp. 1						
I	NNK *ip *+ water	15	25.7 ± 1.83	93.3	2.13 ± 1.19	1.29 ± 0.25
II	NNK *ip *+ GB	15	23.4 ± 1.59	66.7	0.93 ± 0.80*	1.14 ± 0.08**
III	NNK *ip *+ Beer	15	26.0 ± 2.68	46.7	0.73 ± 0.88*	1.19 ± 0.18
IV	saline *ip* + water	12	24.2 ± 4.09	0	0	0
V	saline *ip* + GB	10	23.8 ± 2.63	0	0	0
VI	saline *ip* + Beer	10	28.1 ± 2.60	0	0	0
Exp. 2						
VII	NNK *ip* + water	15	25.3 ± 3.96	100	5.47 ± 1.60	1.44 ± 0.32
VIII	NNK *ip* + NAB (A)	15	26.7 ± 3.40	86.7	3.00 ± 2.36^#^	1.42 ± 0.28
IX	NNK *ip* + NAB (B)	15	26.4 ± 2.44	80.0	1.67 ± 1.40^#^	1.41 ± 0.27
X	saline *ip* + water	10	32.4 ± 3.12	0	0	0
XI	saline *ip* + NAB (A)	10	31.7 ± 2.81	0	0	0
XII	saline *ip* + NAB (B)	10	31.3 ± 1.62	0	0	0

^*^P < 0.005, significantly different from Group I

^**^P < 0.05, significantly different from Group I

^#^*P* < 0.005, significantly different from Group VII

In experiment 2, the average body weights of mice in groups VII–IX were significantly lower than those of mice in groups X–XII (Table 2). No significant differences in the average body weight of the mice were found between groups VII–IX, and X–XII. NNK-injected mice in group VII (PC) developed a significant number of nodules on the surface of their lungs (5.47 ± 1.54, significantly different from group X, *P* < 0.05) (Table 2). The number of nodules per mouse (3.00 ± 2.28) in group VIII, which was administrated NNK and NAB(A), was significantly reduced compared to that of group VII. The number of nodules per mouse (1.67 ± 1.35) in group IX, which was administrated NNK and NAB(B), was also significantly decreased compared to that in group VII. None of the mice in groups X–XII developed lung nodules following the oral administration of NAB(A) or NAB(B).

**Fig. 4:**
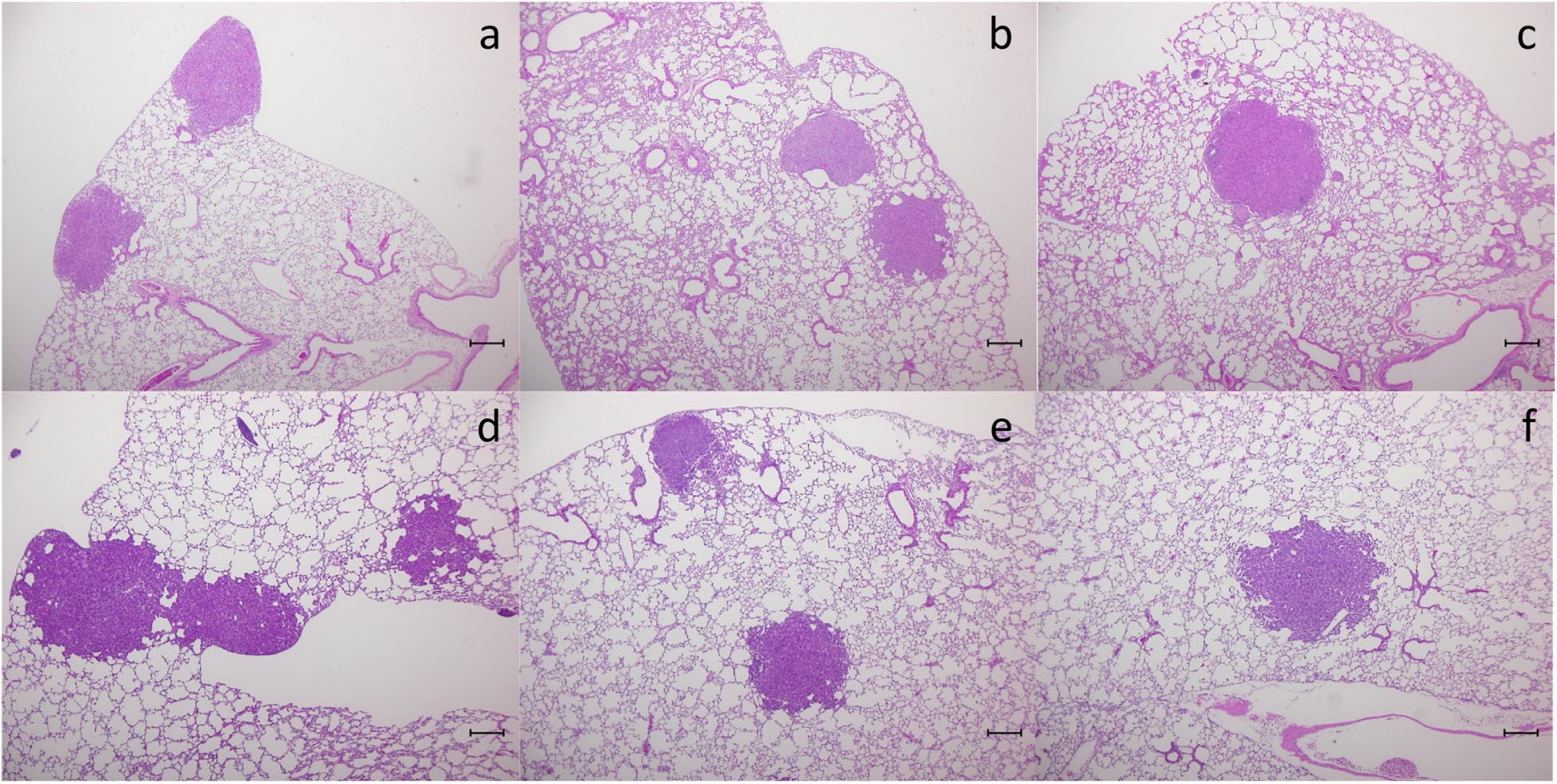
A representative tumor (adenoma/adenocarcinoma) corresponding to the nodule counted microscopically and the alveolar area around the tumor in the A/J mice at 30 weeks of age treated with 4-(methylnitrosamino)-1-(3-pyridyl)-1-butanone (NNK) alone (group I, **a**; group VII, **d**), NNK + GB (group II, **b**), NNK + beer (group III, 4**c**), NNK + NAB(A) (group VIII, **e**) and NNK + NAB(B) (group IX, **f**). GB: glycine betaine; NAB: nonalcoholic beer

This NNK-induced tumorigenesis model is a useful system for detecting the chemopreventive effects of NNK on lung tumorigenesis [23, 25]. The histology of the lungs in A/J mice at 30 weeks of age treated with NNK alone (groups I and VII), NNK + GB (group II), NNK + beer (group III), NNK + NAB(A) (group VIII), and NNK + NAB(B) (group IX) was investigated (Fig. 4). Solid nodules with a maximum diameter of 0.300 mm or more were considered as tumors (adenoma/adenocarcinoma).

### Cell viability

**Fig. 5:**
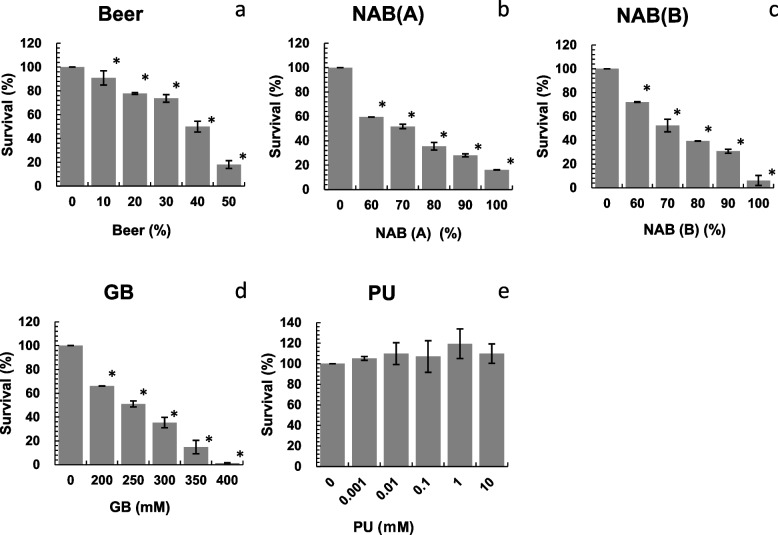
Effects of beer (**a**), NAB(A) (**b**), NAB(B) (**c**), GB (**d**) and PU (**e**) on A549 cell viability. The experiment was repeated thrice and standard deviation (SD) is indicated with the bar (*n* = 5). *Significantly different from each negative control (beer, NAB(A), NAB(B), GB or PU = 0) at *P* < 0.05. NAB: nonalcoholic beer; GB: glycine betaine; PU: pseudouridine

To determine the cytotoxicity of beer, NABs, GB, and PU, A549 cells were treated with the samples at various concentrations, and cell viability was evaluated using the MTT assay. The viability of cells treated with beer decreased in a concentration-dependent manner of 50.0% for 40%beer (Fig. 5a). The viability of cells treated with nonalcoholic beer was 51.8% for NAB(A) and 52.3% for NAB(B) at a concentration of 70%NAB (Fig. 5b and c, respectively). The viability of the cells treated with GB was 51.0% at 250 mM GB (Fig. 5d). In the presence of PU, cell viability did not decrease up to 10 mM of PU (Fig. 5e).

Thus, 40%beer, 70%NAB, 250 mM GB, and 1 mM PU were used for further experiments.

### Effect of beer, NABs, GB and PU on STAT3 and Akt phosphorylation in A549 cells

Overexpression and mutation of the epidermal growth factor receptor (EGFR) are associated with tumor development [26]. We investigated the effects of beer, NAB, GB, and PU on STAT3 and Akt signal transduction.

**Fig. 6:**
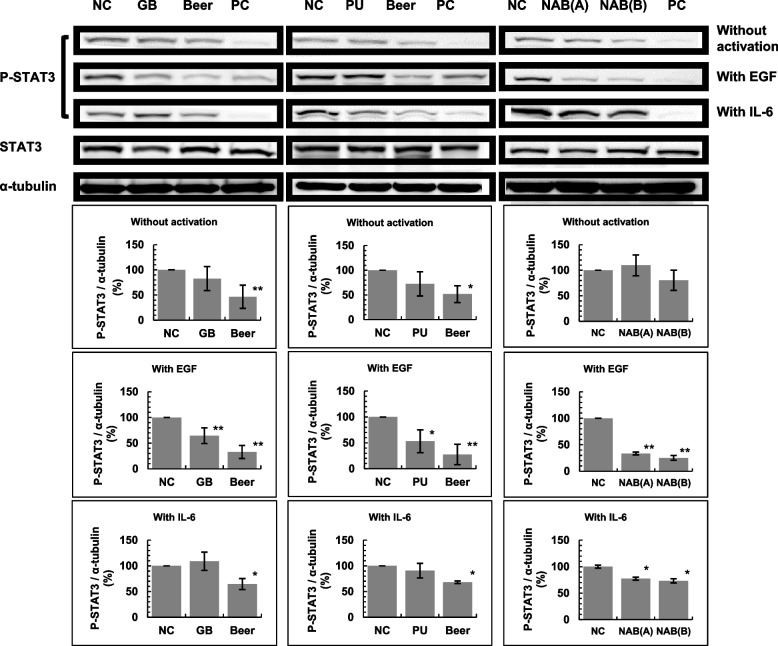
Effects of beer, GB, PU and NABs on STAT3 phosphorylation with or without EGF or IL-6 stimulation in A549 cells. The experiment was repeated thrice and standard deviation (SD) is indicated with the bar (*n* = 3). *Significantly different from the negative control (NC; beer = 0, GB = 0, PU = O and NABs = 0) at *P* < 0.05. *Positive control (PC), Stattic (final 20 µM) serving as a positive kinase-inhibitor control was added to the PC dish 1 h prior to the termination of incubation. NAB: nonalcoholic beer; GB: glycine betaine; PU: pseudouridine; EGF: epidermal growth factor; IL-6: interleukin 6

The phosphorylation of STAT3 in A549 cells treated with 40%beer, 70%NABs, 1 mM PU, and 250 mM GB was examined. Without EGF or IL-6 stimulation, the phosphorylation of STAT3 was inhibited in the presence of beer (Fig. 6). Upon EGF activation, the phosphorylation of STAT3 was significantly inhibited by beer, GB, PU, and NABs. The STAT3 pathway is also activated by IL-6 stimulation as a JAK/STAT signaling pathway [27] and the effect of IL-6 stimulation was also investigated. Upon IL-6 stimulation, a significant inhibition of STAT3 phosphorylation was observed in the presence of beer and NABs. Therefore, all these samples inhibited EGFR signaling pathways via STAT3.

**Fig.7:**
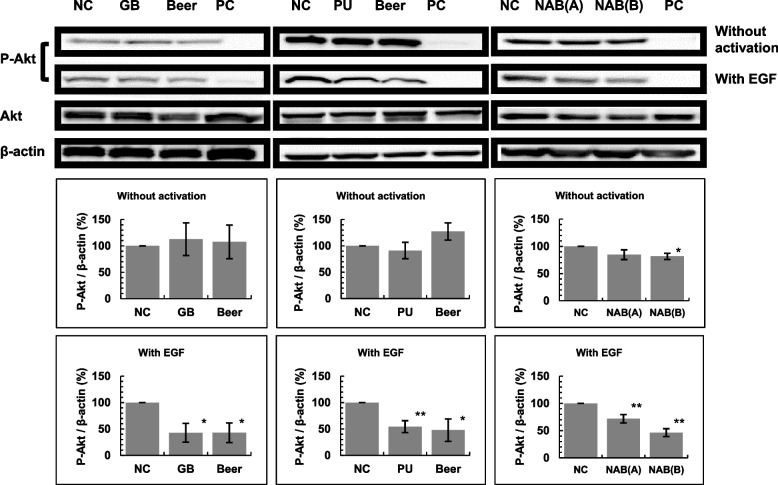
Effects of beer, GB, PU and NABs on Akt phosphorylation with or without EGF stimulation in A549 cells. The experiment was repeated thrice and standard deviation (SD) is indicated with the bar (*n* = 3). *Significantly different from the negative control (NC; beer = 0, GB = 0, PU = O and NABs = 0) at *P* < 0.05. Positive control (PC), LY294002 (final 50 µM) serving as a positive kinase-inhibitor control was added to the PC dish 1 h prior to the termination of incubation. NAB: nonalcoholic beer; GB: glycine betaine; PU: pseudouridine; EGF: epidermal growth factor

We investigated the effects on the PI3K/Akt/mTOR pathway, especially, phosphorylation of Akt by beer, NABs, and beer components (PU and GB) (Fig. 7). Without EGF stimulation, Akt phosphorylation is inhibited in the presence of NAB(B). Upon EGF activation, Akt phosphorylation was significantly inhibited by beer, GB, PU, and NABs.

## Discussion

Beer has been reported to exert various biological effects, including cancer chemoprevention [13]. As awareness of the effects of ethanol on the human body and an increase in healthy lifestyle habits [14], we focused on the biological activities of NABs. There are various NABs with special processing and differences in raw materials, tastes, and flavors (Table 1). A comparative study of the biological activities, especially the antimutagenicity and antitumorigenicity, of beer and NABs is worth investigating.

Carcinogenesis is the process whereby a normal cell is transformed into a neoplastic cell, and involves several steps starting with initiation, followed by promotion and progression [28]. Mutations in the driver genes are necessary for carcinogenesis. We hypothesized that beer, NABs, and beer components would inhibit the mutagenicity of the alkylating agent, MNNG and NNK. As shown in Figs. 1 and 2 (circles), beer, NABs, LAB, GB, and PU inhibited the mutagenicity of MNNG in *S. typhimurium* TA1535. We investigated the differences in antimutagenicity between beer, LAB, and five NABs. The ID_50_ for the antimutagenicity of beer (0.5 µL/plate) was lowest and those of NABs and LAB toward MNNG ranged from 1.5 µL (NAB(B)) to 15.8 µL (NAB(F)) (Fig. 1, Table 1). ID_50_ of NAB(B) was nearly 10 times fewer than ID_50_ of NAB(F). These differences in the antimutagenic effects on MNNG may be due to the variety of raw materials, ingredients, and components in each drink (Table 1). Beer, NABs, LAB, GB, and PU also inhibited the mutagenicity of NNK in *S. typhimurium* TA1535 (Fig. 3, circle). In contrast to the results obtained using *S. typhimurium* TA1535, the mutagenicity of MNNG and NNK detected in *S. typhimurium* YG7108 did not decrease with beer, NABs, GB, or PU (Figs. 2 and 3, square). This suggests that the antimutagenic effects of beer, NABs, GB, and PU are mediated by ogt_ST_ and ada_ST_ enhanced DNA damage repair. They may enhance *O*^6^-methylguanine DNA methyltransferases in *S. typhimurium* TA1535 to decrease the mutagenicity detected with *S. typhimurium* TA1535. Therefore, the mutagenicity detected in *S. typhimurium* YG7108 (lacking *O*^6^-methylguanine DNA methyltransferases) was not suppressed by beer, NABs, GB, or PU (Figs. 2 and 3, squares).

The GB content was 92 µM and 290 µM in NAB (A) and NAB(B), respectively, and the PU content was 5.2 µM and 22 µM in NAB (A) and NAB(B), respectively. As GB contents in beer was reported as 7.00 mg/100 mL (0.600 mM) [15], and PU content in beer was reported as 0.432 mg in 100 mL beer, that is 17.7 µM [16], GB and PU contents in NABs were comparable to those in beer. The ID_50_ of GB on MNNG mutagenicity was 0.220 mmol/plate, whereas the amount of GB in the ID_50_ volume of NAB(A) on MNNG mutagenicity (3.1 µL/plate) was calculated to be 285 pmol/plate, which is 0.13% of the ID_50_, and the amount of GB in the ID_50_ of NAB(B) (1.6 µL/plate) was calculated to be 464 pmol/plate, which is 0.21% of the ID_50_. ID_50_ of PU on MNNG mutagenicity was 0.50 µmol/plate, whereas the amount of PU in ID_50_ volume of NAB(A) on MNNG mutagenicity (3.1 µL/plate) was calculated to be 16.1 pmol/plate, that is 3.2% of ID_50_, and the amount of PU in ID_50_ of NAB(B) (1.6 µL/plate) was calculated to be 35.2 pmol/plate, that is 7.0% of ID_50_. The contribution ratios of GB and PU to the antimutagenicity of NABs were comparable to those in beer (0.14% and 1.8%, respectively), as previously described [15, 16]. The contribution ratios of GB and PU in the NAB samples accounted for 7% or less of the total antimutagenicity of NAB. Thus, other active components in NAB remain to be identified, and the active components in NAB may additively or synergistically contribute to its antimutagenicity.

We investigated the antitumorigenic activity of beer, NAB(A), NAB(B), and GB on NNK-induced lung tumorigenesis in vivo. Unfortunately, PU is too expensive for in vivo experiments. NNK-induced lung nodule formation was significantly reduced (Table 2). More than 50% of the mice in the NNK + beer group (group III) and 33% in the NNK + GB group (group II) had no nodules in their lungs (Table 2). The average nodule size in the NNK + GB group (group II) was significantly smaller than that in the PC group (group I).

During multistep carcinogenesis, the suppression of cancer cell growth is an important target for anti-tumorigenesis. These samples may suppress the growth of cancer cells to form countable nodules. Tumor nodules > 1 mm in diameter were counted, and the number of nodules > 1 mm in diameter was significantly decreased in mice that received beer, NABs, or GB (Table 2). We investigated whether there was an inhibitory effect on cancer cell growth. Driver gene mutations have an important role in cancer cell growth [29], and it is known that many patients with lung adenocarcinoma have driver gene mutations. In Japan, the driver gene abnormalities in non-small cell carcinoma, which is the most common of the EGFR gene abnormalities, is reported as being due to a mutation found in 35% of patients with lung adenocarcinoma [30, 31]. Therefore, we investigated whether beer, NABs, and beer components inhibit the EGFR signaling pathway. EGFR signaling was mediated with several dawn-stream pathways, especially Akt and STAT3 [32–34]. Phosphorylation of Akt and Stat3 in human-derived A549 cells significantly decreased in the presence of beer, NABs, GB, or PU with EGF stimulation (Figs. 6 and 7). The STAT3 pathway is also activated by IL-6 stimulation via the JAK-STAT signaling pathway [32, 33]. Beer, NABs, GB, and PU also suppressed the IL-6 mediated JAK/STAT signaling pathways (Fig. 6). Our results showed that the phosphorylation of Akt and Stat3 in human-derived A549 cells was significantly decreased in the presence of beer, NABs, or beer components with EGF or IL6 stimulation, indicating that the components in beer or NABs suppressed the PI3K/AKT and JAK/STAT signaling pathways (Figs. 6 and 7).

The concentration of GB in the 40%beer, 70%NAB(A) and 70%NAB(B) was calculated as 239 µM, 64.4 µM and 203 µM, respectively, and the concentration of PU in the 40%beer, 70%NAB(A) and 70%NAB(B) was calculated as 7.1 µM, 3.64 µM and 15.4 µM, respectively. STAT3 and Akt phosphorylation experiments were performed using 1 mM PU or 250 mM GB. Therefore, the amounts of PU and GB in beer and NABs may be insufficient to suppress Akt and STAT3 phosphorylation. Other components of beer and NABs, along with GB and PU, may also suppress the phosphorylation of Akt and STAT3 in A549 cells.

The raw materials of beer and NABs are malts, hops and other ingredients (Table 1), and components, including GB and PU may be produced from such raw materials or fermentation processes. It will be interesting to note that daily consumption of drinks, including beer and NABs, may have the potential for cancer prevention. PU exists in higher organisms as a minor base; however, little is known about its physiological activity. Nucleoside modification in RNA, from U to PU, was reported to change the immune response of dendritic cells [35], but there have been few reports on the monomer's function of PU. In this study, we found that PU affects signal transduction in A549 cells.

In conclusion, beer, NABs, and GB reduced the NNK-induced lung tumorigenesis. They target both the initiation and growth/progression steps of carcinogenesis, specifically via antimutagenesis, stimulation of alkyl DNA adduct repair, and suppression of Akt- and STAT3- mediated growth signaling. GB and PU may contribute, in part, to the biological effects of beer and NABs via antimutagenicity and the suppression of Akt and STAT3 phosphorylation.

## Data Availability

Not applicable.

## Abbreviations

DMEM: Dulbecco's Modified Eagle Medium
EGF: Epidermal growth factor
EGFR: Epidermal growth factor receptor
GB: Glycine betaine
HE: Hematoxyilin and eosin
i.p.: Intraperitoneal
IL-6: Interleukin 6
LAB: Low alcoholic beer
MNNG: 1-Methyl-3-nitro-1-nitrosoguanidine
MTT: 3-(4,5-Dimethyl-2-thiazolyl)-2,5-diphenyl-2H-tetrazolium bromide
NAB: Nonalcoholic beer
NABs: Nonalcoholic beers
NC: Negative control
NNK: 4-(Methylnitrosamino)-1-(3-pyridyl)-1-butanone
PC: Positive control
PhIP: 2-Amino-1-methyl-6-phenylimidazo[4,5-b]pyridine
PU: Pseudouridine
SD: Standard deviation
S9: Supernatant fraction of rat liver homogenate
TBST: Tris-buffered saline containing 0.1% Tween 20
